# Detection and Recognition of Bilingual Urdu and English Text in Natural Scene Images Using a Convolutional Neural Network–Recurrent Neural Network Combination with a Connectionist Temporal Classification Decoder

**DOI:** 10.3390/s25165133

**Published:** 2025-08-19

**Authors:** Khadija Tul Kubra, Muhammad Umair, Muhammad Zubair, Muhammad Tahir Naseem, Chan-Su Lee

**Affiliations:** 1 Faculty of Information Technology and Computer Science, University of Central Punjab, Lahore 54000, Pakistan; l1f21mscs0040@ucp.edu.pk (K.T.K.); muhammad.umair@ucp.edu.pk (M.U.); 2 Interdisciplinary Research Center for Finance and Digital Economy, King Fahd University of Petroleum and Minerals, Dhahran 31261, Saudi Arabia; muhammad.zubair@kfupm.edu.sa; 3 Department of Electronic Engineering, Yeungnam University, Gyeongsan-si 38541, Republic of Korea

**Keywords:** convolutional neural network, recurrent neural network, bidirectional long short-term memory, bidirectional gated recurrent unit, connectionist temporal classification, convolutional recurrent neural network, natural scene images, multilingual, bilingual, text recognition

## Abstract

Urdu and English are widely used for visual text communications worldwide in public spaces such as signboards and navigation boards. Text in such natural scenes contains useful information for modern-era applications such as language translation for foreign visitors, robot navigation, and autonomous vehicles, highlighting the importance of extracting these texts. Previous studies focused on Urdu alone or printed text pasted manually on images and lacked sufficiently large datasets for effective model training. Herein, a pipeline for Urdu and English (bilingual) text detection and recognition in complex natural scene images is proposed. Additionally, a unilingual dataset is converted into a bilingual dataset and augmented using various techniques. For implementations, a customized convolutional neural network is used for feature extraction, a recurrent neural network (RNN) is used for feature learning, and connectionist temporal classification (CTC) is employed for text recognition. Experiments are conducted using different RNNs and hidden units, which yield satisfactory results. Ablation studies are performed on the two best models by eliminating model components. The proposed pipeline is also compared to existing text detection and recognition methods. The proposed models achieved average accuracies of 98.5% for Urdu character recognition, 97.2% for Urdu word recognition, and 99.2% for English character recognition.

## 1. Introduction

Language diversity is prevalent around the globe [1], and approximately 6800 spoken languages and 300 writing systems are used in multilingual nations [2]. These languages symbolize the ethnolinguistic identity of individuals [3]. Urdu and English are highly dominant and widely spoken in the Middle East (Bahrain, Oman, Qatar, Saudi Arabia, and the UAE); Southern and Eastern Africa (Botswana, Malawi, Mauritius, South Africa, and Zambia); Europe (Germany, Norway, and the UK); South America (Guyana); and particularly in South Asia (Afghanistan, Bangladesh, Nepal, India, and Pakistan), including other countries such as Fiji and Thailand [4]. English and Urdu are also official languages of Pakistan [5,6]. These languages are widely used globally across sectors such as government, law, banking, education, and business (for import and export), primarily because they facilitate efficient communication [7].

Visual text communication is used in public spaces, particularly on signboards, navigation boards, hoardings, and banners, offering useful information and guidelines. Humans rely on this semantic textual information to effectively interact with their surroundings. A recent study reported that individuals focused more on the text when they were shown an image containing text and nontext objects [8], indicating that text recognition is crucial for understanding a natural scene image (NSI).

If text in NSIs can be accurately detected and recognized, it can be useful for various applications such as real-time translation, political poster identification, advertisements, video indexing, scene understanding, robot navigation, and industrial automation [9]. In particular, autonomous driving systems will benefit from such text recognition. Autonomous vehicles are anticipated to be the future of transportation [10], indicating the importance of detecting text in NSIs for achieving autonomous driving.

**Figure 1:**
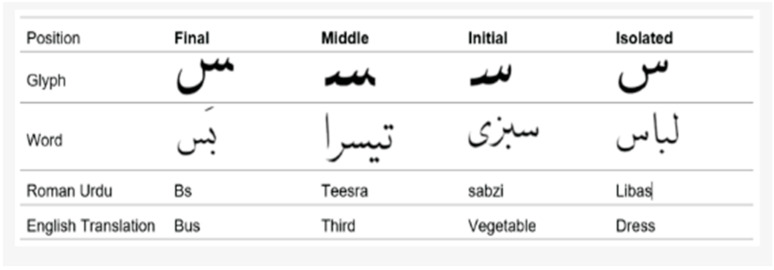
Multiple forms of the same Urdu character in different positions in a word [12].

In this study of bilingual NSIs, one of the languages analyzed is Urdu, which is a complex language because it is written in cursive [11]. Multiple forms of the same character in Urdu must be differentiated from one another [12]. Each character takes a different shape depending on its position in a word. Figure 1 shows that when “Seen” is written at the end of the words “Libas” and “Bs,” it takes a particular shape. However, when it is written at the beginning and center of the words “Sabzi” and “Teesra,” respectively, it takes different shapes [12].

**Figure 2:**
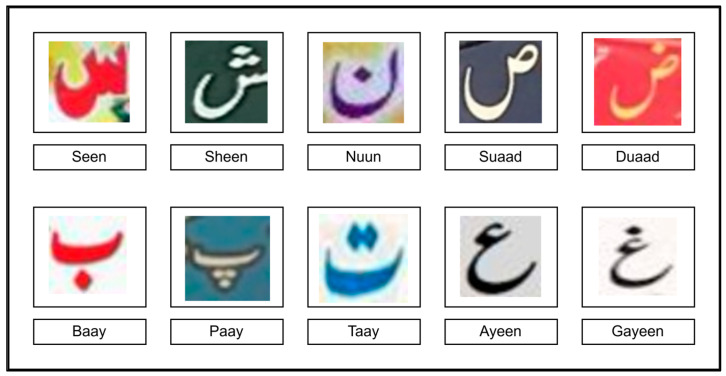
Various Urdu characters from a publicly available dataset [13] and their cursive appearance. Many characters have the same base components that resemble other characters and are differentiated only by the number and placement of dots on them. Some samples contain natural blur, as present in the original dataset, reflecting real-world NSI conditions like blur or lighting effects.

Many Urdu characters share similar base shapes. As shown in Figure 2, “seen” can resemble other characters such as “Sheen,” “Nuun,” “Suaad,” and “Duaad.” Similarly, “Baay” resembles “Paay” and “Taay,” and “Ayeen” resembles “Gayeen”.

Some Urdu characters can only be differentiated based on the number of dots on them. As in Figure 2, although “Seen” and “Sheen” resemble each other, the former contains two dots and the latter contains three dots. Similarly, “Suaad” has no dots but exactly the same shape as “Duaad” with one dot. “Ayeen” has no dots but has the same shape as “Gayeen” with one dot. Some Urdu characters can also be differentiated by the placement of dots around them, such as “Baay” and “Paay” which have dots placed below them, whereas “Taay” has dots above it (Figure 2). These differences make it difficult to recognize Urdu characters.

Figure 2 shows different cropped images of Urdu characters from a publicly available dataset [13], illustrating its cursive font. Also, a few character samples in Figure 2 appear slightly blurred, which is intentional to simulate real-world NSI challenges where text may be captured in motion or under suboptimal conditions.

**Figure 3:**
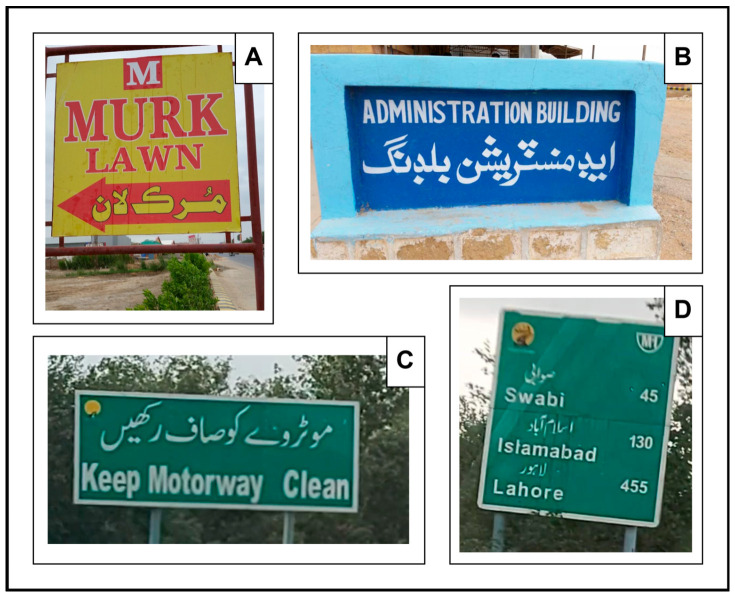
Samples of bilingual NSIs obtained from a publicly available dataset [13]: (**A**) Urdu word “Speed” showing character connectivity; (**B**) Urdu word “administration” showing syllable connectivity; (**C**) English text with non-cursive characters; (**D**) Different orientations of Urdu and English text. All samples highlight the differences in Urdu and English text in NSIs.

English is another language used in bilingual NSIs and is different from Urdu. It is written with a left-to-right orientation [14] contrary to Urdu with a right-to-left orientation [15], as shown in Figure 3. In addition, English is a non-cursive language [16], while Urdu is a cursive language, i.e., Urdu characters in a word are interconnected [17], whereas English characters are not [18]. For example, Figure 3 image “B” shows character connectivity in syllables of the Urdu word “administration”, that is “ad” “ministr” “ation”. Another example in Figure 3 image “A” shows character connectivity in the whole Urdu words “Speed” and “Checking”. In contrast, the characters in the English words and syllables are not interconnected. Thus, differentiating between English and Urdu in NSIs becomes challenging.

Figure 3 shows different samples of bilingual NSIs from the publicly available dataset [13], illustrating the distinct nature of Urdu and English.

**Figure 4:**
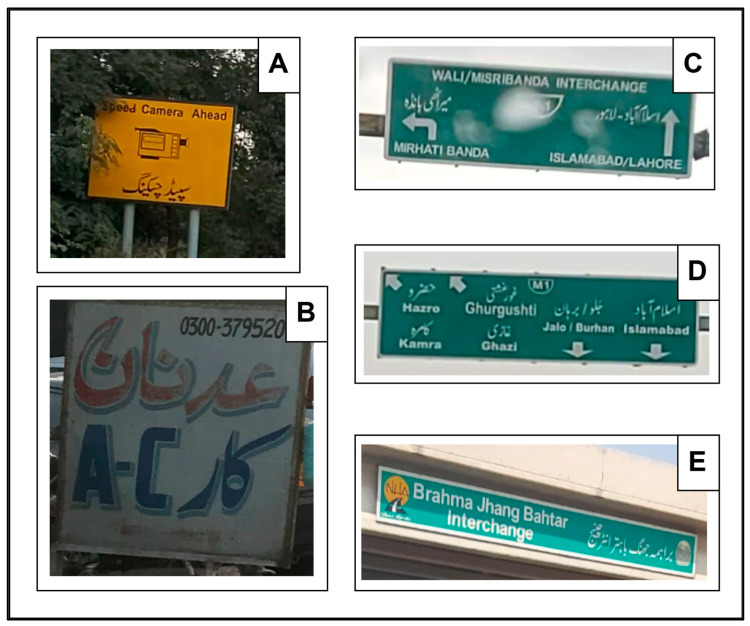
Samples of NSIs from a publicly available dataset [13]: (**A**) NSI with nontext regions and objects; (**B**) NSI with low brightness and varying fonts; (**C**) Rainy day causing reduced quality and blurriness; (**D**) Blurred NSI due to distance; (**E**) Tilted signboard with different text orientation. All samples illustrate factors influencing text detection and recognition tasks in NSIs.

In addition, various factors such as brightness, blur, font types, background complexity, opacity, size, texturing, orientation, and nontext regions and objects [19] in NSIs add to the complexity of text detection and recognition tasks (Figure 4).

Figure 4 reveals a shadow is in images “B” and “E” due to the brightness level, reducing the quality of text in both images. Compared to other images in Figure 4, image “B” has very low brightness because it was captured in the evening. This indicates that lighting can impact the performance of text recognition tasks in NSIs. Weather conditions can also impact the NSI quality. For instance in Figure 4, the image “C” was taken on a rainy day, and the quality of text in this image is low due to water droplets. The distance and height of signboards are directly related to image blurriness. For instance in Figure 4, images “C” and “D” were taken from a distance and they therefore appear blurred.

NSIs may contain different text fonts within the same language. For example, in Figure 4, images “B” and “E” display Urdu and English text in varying font styles. NSIs may also feature text with different textures, as shown in all images of Figure 4, particularly in image “B”. Moreover, Image “E” of Figure 4 has a different text orientation because of the tilted position of the signboard. Image “A” of Figure 4 is an ideal NSI because it contains nontext regions and objects. For instance, a camera icon is seen between text, which is a nontext object, and leaves are present on the word “Speed,” which is a nontext object. The surrounding leaves on image “A” in Figure 4 are a nontext region. All images of Figure 4 have highly complex backgrounds, such as the leaves in the background of image “A”, whereas there are clouds in the background of images “C” and “D”. Images “B” and “E” also have objects in their backgrounds. Figure 4 presents various samples of NSIs from the publicly available dataset [13], illustrating factors that influence text detection and recognition tasks in natural scene images.

**Figure 5:**
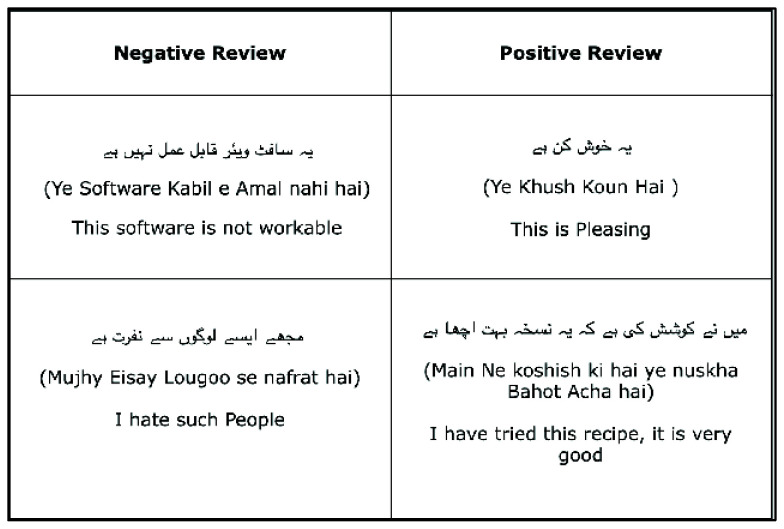
Scanned document image [20] with plain white background containing uniformly typed Urdu and English text.

These adverse factors (such as brightness, blur, font types, background complexity, etc.) of NSIs cannot be seen in scanned document images. Figure 5 shows that scanned document images tend to have a simple background contrary to NSIs with complex backgrounds. The backgrounds of NSIs are nonuniform and contain various sceneries, whereas scanned document images typically have a white background with a black font text. NSIs have various text textures such as typed, handwritten, and painted, whereas scanned document images generally have a typed text texture (Figure 5).

NSIs also have various nontext objects and regions. The scanned document image in Figure 5 shows that these documents have a lower tendency to contain nontext objects and regions compared to that of NSIs. Thus, recognizing text in NSIs is a very challenging task. Figure 5 shows a scanned document image [20].

Text detection and recognition have garnered considerable research attention for complex NSIs in recent years. Several studies have addressed these tasks for Urdu and English text. For instance, a previous study on Urdu text detection [21] achieved good recognition results; however the NSIs in their work contained manually pasted text. Similarly, many studies have been conducted for the detection and recognition of text in NSIs [22,23,24,25]. However, these studies were unilingual and detected only Urdu characters in bilingual NSIs obtained from a publicly available dataset [13]; this dataset has been used in unilingual studies such as Urdu. In another unilingual text recognition study [26,27], however, only English text was detected and recognized. Various other studies analyzed both Urdu and Arabic text [28], as well as [29] Urdu and English text; however, they used very small datasets and did not detect text in images with different orientations and contexts. Dataset size plays a key role in the performance of convolutional neural network (CNN) models, and the models trained on smaller datasets show a strong tendency of overfitting [30,31]. This indicates that the larger the data, the higher the model accuracy. Another study performed bilingual text recognition [32] using artificial neural networks but achieved less accuracy than the proposed model. Therefore, a large dataset must be constructed for detecting text in NSIs containing both Urdu and English texts. A bilingual mechanism for the detection and recognition of these texts must also be developed.

Herein, a pipeline for Urdu and English (bilingual) text detection and recognition in complex NSIs is proposed. A bilingual dataset was constructed for training this pipeline, and experiments were conducted using different recurrent neural networks (RNNs) and hidden units in RNNs to identify a suitable RNN for the tasks. Ablation studies were conducted on the two best models to highlight the importance of their components. Finally, these models were compared with existing models (HOG, CNN, and MLFF) used for text detection and recognition in NSIs.

The remainder of this paper is organized as follows: Section 2 describes the state-of-the art methods used for text detection and recognition. Section 3 discusses the limitations of previous studies and the contributions of this study. Section 4 describes the dataset used and its preprocessing. Additionally, the proposed text detection and recognition method is discussed in detail. Section 5 discusses the evaluation indicators and their significance. Section 6 covers the experiments conducted and their corresponding results. Section 7 concludes the paper and discusses future works.

## 2. Related Works

Text detection and recognition have undergone significant progress with advancements in machine learning and neural networks. These tasks are crucial for understanding image–text relations. A comprehensive discussion on these topics is presented in the subsequent sections.

### 2.1. Text Detection

During the early days of machine learning, text detection relied on handcrafted features. For instance, Hossain et al. proposed Maximally, which is a method used for signboard text recognition in traffic boards using maximally stable extremal regions (MSERs) [33]. Such texts usually have a high contrast against their background. This method analyzes the image to identify potential text regions based on their intensity. It uses the MSER algorithm to detect text regions that remain consistent across various threshold levels. Basavaraju developed a method based on Laplacian component fusion analysis for bilingual text detection from images and videos [34]. Although inventive, this method was highly sensitive to lighting scenarios and yielded unpredictable and variable text detection results. It also faced various challenges, particularly in detecting text under varying luminous conditions that worsened the quality of the detected text. It yielded low accuracy, particularly in real-time applications that required rapid processing.

Tian et al. proposed a co-occurrence histogram of oriented gradient (CoHOG) and convolutional CoHOG feature descriptors based on scene text detection [35]. To detect text regions from an input image, CoHOG incorporates context-aware spatial information in neighbor pixels. These methods, however, require a series of manually performed feature extraction steps and modifications of classifiers; thus, they are structurally complex and employ time-consuming processes. The advancements in computer vision have facilitated the implementation of end-to-end representation learning-based methods for text detection.

Liao developed a network that focused on differentiable binarization (DB) to process and segment images [36]. This methodology was implemented by converting the images into a binary format and creating a contrast between the image and text. The quality of the results considerably depended on DB. However, this method cannot accurately divide images containing complex background and nontext objects into binary pixels. Therefore, the network was considerably modified to perform well across different image types. Saha proposed a text detection method [37] that combined stroke width transform (SWT) and MSERs specifically for outdoor text images. It used SWT to identify text regions, enabling the differentiation of text from other components. Then, MSERs were used to identify more precise text regions. However, SWT did not offer high performance for text with irregular stroke widths or heavy distortions. Additionally, MSERs struggled with complex backgrounds or overlapping text. However, these methods which only focused on unilingual text detection had limited applicability.

### 2.2. Text Recognition

Bains developed a mechanism for detecting text in signboards, particularly applied in Gurumukhi [38]. It used convolutional neural networks for training from a dataset containing signboard images. The CNN was well trained to extract, learn, and recognize text from images. Aberdam presented an attention-driven technique, known as sequence-based contrast learning (SeqCLR), for text recognition [39]. This technique employed a sequence model that was trained to predict text sequences from images via an attention mechanism. This technique enabled the model to analyze different regions in an image based on their weight to the text being recognized. SeqCLR refined the ability of the model to differentiate between different text components. However, it was highly sensitive to minor shifts or drifts in the attention mechanism. Even minor drifts in the distribution of attention can considerably impact the model performance. To address this issue, Cheng developed a mechanism based on the residual network (ResNet) architecture [40]. This architecture was well trained for complex patterns. The focusing attention network has also been studied and focuses on text in minor shifts and drifts. This combined methodology is useful for detecting text in areas hindered by lighting conditions, orientations, and complex backgrounds.

Lu proposed a methodology to improve text recognition in NSIs using a transformer-based approach [41] that could powerfully handle sequential data. This method could handle text images of various lighting conditions and patterns along with text of various styles, sizes, and shapes. Although this method had many strengths, it had limitations when dealing with images of curvature distortions and orientations. To address this limitation, Shi proposed a unified network-based methodology for parallel scene text recognition [42] and detection. Thus, it required a single framework unit rather than a pipeline, making it particularly useful for conditions with distortions in text.

Chen also proposed a method to improve text recognition [43] that was based on transformers integrated with attention-driven and rectification mechanisms. This method could efficiently deal with various text appearances in real-world examples. Busta proposed a deep CNN-based method for the recognition of multilingual text [44]. This approach used a single framework for detecting and recognizing text in parallel, thereby enhancing the detection accuracy and timing. A fully connected network was introduced to extract and learn patterns from text in NSIs.

### 2.3. Urdu Dataset and Research

Methods used for the detection and recognition of cursive Urdu text have made significant progress. Several studies have attempted to improve the accuracy and enhance the model performance to deal with such texts. Arafat and Iqbal employed deep learning for the detection and recognition of Urdu text in NSIs [21] and proposed a method that facilitated the detection, orientation prediction, and recognition of Urdu ligatures in outdoor images. A custom fast region-based convolutional neural network (RCNN) algorithm was used with popular CNNs such as Squeezenet, Googlenet, Resnet18, and Resnet50 for image detection and localization. For predicting ligature orientation, a custom regression residual neural network was trained and tested on datasets containing randomly oriented ligatures. Ligatures were recognized using a two-stream deep neural network. Finally, all four detectors were evaluated and compared for their ability to detect/localize Urdu using average precision. However, these studies performed the aforementioned tasks by pasting Urdu text on images manually.

Chandio used the HOG method [22] that was specially designed to extract the features of the detected text. It analyzed the values of gradients in the text and backgrounds and easily segregated the background from the text. After feature extraction, five different classifiers were used to learn them, such as the support vector machine (SVM), k nearest neighbor (kNN), random forest classifier, extra tree classifier, and multilayer perceptron (MLP). Ali [23] used a simple CNN architecture to extract, analyze, and learn Urdu text and used a Softmax function to classify the text. Both these contributions were for unilingual text and yielded lower accuracy than the proposed model.

Leghari used a multilevel deep neural network for recognizing Urdu characters in NSIs [24]. This network used a fusion of CNNs to recognize text such as multiscale feature aggregation (MSFA) and Multilevel Feature Fusion (MLFF). This method focused on the image at different levels, and deep-level features were extracted and added to the abstract-level features of the CNN layer. Thus, the fusion of CNNs enhanced the recognition accuracy. In addition, Pickering [25] employed a convolutional RNN for detecting and recognizing both Urdu characters and words; however, it yielded lower recognition rates than the proposed model.

A dataset [13] for Urdu text detection is publicly available and contains bilingual NSIs of Urdu and English text as the base segment. It also contains images of cropped Urdu characters and words. This dataset is widely used by researchers working on Urdu text due to its structured and well-labeled format. Studies on Urdu text recognition have made significant progress; however, the proposed model outperformed on this dataset [13] compared to the aforementioned models [22,23,24,25]. Prior text recognition methods used traditional machine learning techniques, such as manual segmentations, handcrafted feature extraction (e.g., HOG in [22]), and conventional classifiers like SVM, kNN, and MLP. These methods are now considered outdated compared to modern deep-learning-based pipelines, particularly in Urdu text recognition. The proposed model incorporated all these factors and introduced a solution pipeline for efficient detection and recognition of text in NSIs.

### 2.4. English Language Dataset and Research

Herein, recent studies solely based on English text detection and recognition in NSIs are discussed. A previous study employed Rank-1 tensor decomposition for English text detection and recognition [26]. It yielded better results compared to the HOG method on publicly available datasets such as Char74 and ICDAR2003. Another study detected and recognized English text [27] in NSIs from two publicly available datasets, Char74K and ICDAR2003, using CNN and KNN.

The proposed model achieved more accurate results compared to these models and is based on the convolutional recurrent neural network (CRNN). A more efficient model for feature extraction of English text was also developed that could handle English and Urdu text. It could thus be employed for detecting text in images with different orientations and texture languages.

### 2.5. Bilingual Text Detection and Recognition

Panhawar proposed a method based on artificial neural networks to detect and recognize text on signboards [32]. It first identified the text regions, followed by text recognition. Artificial neural networks rely on the quality and quantity of data used for training and are not as robust as CNNs. Artificial neural networks cannot handle data of different styles and orientations, and NSIs contain various data types including styles, textures, and orientations. Therefore, these networks cannot be employed for detecting and recognizing text in NSIs. Convolutional feature fusion has also been used for detecting text in NSIs [28]. Herein, a feature fusion of convolutional layers in a VGG-16 network was employed to detect bilingual text in NSIs. A fast RCNN was employed to produce text/nontext proposals, which were then fed to the RNN to link them in sequence. The RNN was embedded within the convolutional network. The proposed network was trained on a combined ICDAR 2017 bilingual NSI text dataset and a custom manually labeled Urdu text dataset. The proposed method achieved satisfactory results for Arabic and Urdu text detection in NSIs.

Butt used the Deep Learning Laboratory’s Traffic Signboard Dataset (DLL-TraffSiD) to develop bilingual text (Arabic and Urdu) detection and recognition methods for traffic signboards [29]. A pipeline was also developed for bilingual text detection and recognition for an outdoor road environment. Results showed that the system had wide applicability in text detection and text recognition on the proposed dataset. However, this method was employed on small datasets and was prone to overfitting [30,31]. Thus, a mechanism was developed for detecting and recognizing Urdu and English text (bilingual) in all types of NSIs from a large dataset. Therefore, a bilingual dataset and pipeline model were developed to address these issues and to classify Urdu and English text from complex NSIs.

## 3. Limitations of Previous Studies and the Contributions of This Study

Despite significant advancements in text detection and recognition, existing methods face several limitations that hinder their applicability in real-world, multilingual, and complex scenarios. These have been discussed in Section 3.1, and our contributions to addressing these limitations and challenges are discussed in Section 3.2.

### 3.1. Limitations of Previous Studies

The limitations of existing methods in text detection and recognition have been summarized below. Table 1 shows the methodology, datasets, and languages used in previous studies and their limitations.

Earlier approaches that relied on handcrafted features or traditional neural networks faced limitations under varying illumination conditions and complex backgrounds [33,37]. Moreover, existing studies predominantly focused on either English or a limited set of languages, offering limited effectiveness in cursive texts [34,36,41,42,43]. Some studies discussed limitations of methods in detecting text with arbitrary orientations [34,35,37], and some reported on the inability of models to detect text in NSIs because their attention mechanism shifted to unrelated or irrelevant areas of an image, leading to errors in text localization or recognition [36,39,40].

Some studies only focused on Urdu and were not robust in dealing with text with multiple orientations [21,22,23,24,25]. Although several studies have focused on multilingual text detection [28], they only focused on cursive text with a single orientation. They lacked robustness when dealing with text with multiple orientations. Additionally, a study focusing on Urdu and English text recognition [29] used a very small dataset, and the developed model was prone to overfitting. Another study on Urdu and English text recognition [32] was based on neural networks and offered very low accuracy. These findings indicate the need to develop a pipeline that can handle bilingual text with different orientations and complex backgrounds, while also avoiding overfitting.

### 3.2. Our Contributions

Herein, current methodologies were extended to provide a more robust and inclusive bilingual mechanism for detecting and recognizing Urdu and English texts while addressing complexities in NSIs.

The diversity in Urdu and English texts and various factors of NSIs make the text recognition and detection tasks challenging. Moreover, the detection of bilingual text in NSIs has not been extensively studied. Therefore, a benchmark dataset of Urdu and English with multiple augmentations and a pipeline for detecting and recognizing these texts in NSIs were developed herein. In particular, we aimed to achieve the following:

- Develop a model that handles cursive Urdu font with various positions and shapes;
- Develop a robust mechanism that addresses challenges associated with the detection and recognition of bilingual text (Urdu and English) with differing script characteristics and orientations;
- Establish a method for detecting text in complex NSIs with varying factors such as brightness, blur, background complexity, and nontext regions and objects;
- Construct a bilingual dataset of Urdu and English text from a preliminary unilingual dataset to address the lack of an adequate dataset featuring NSIs with Urdu and English text;
- Create a pipeline for accurate text detection and recognition using a CNN, an RNN, and a connectionist temporal classification (CTC).
  ◦ The proposed pipeline used a customized CNN for feature extraction.
  ◦ It was evaluated using different types of RNNs and hidden units in RNNs.
  ◦ The best models derived using this pipeline were subjected to ablation studies.

This pipeline was compared with existing methods used for text detection and recognition and evaluated on the publicly available Cursive Text dataset [13] to ensure fair and direct comparison.

## 4. Materials and Methods

Section 4.1 discusses the datasets and their preparation for subsequent analysis, and Section 4.2 explains the proposed methodology.

### 4.1. Materials

The characteristics and segments of the dataset used in this study are discussed herein along with the conversion of a unilingual dataset into a bilingual dataset. The augmentation and preprocessing techniques used to enhance the dataset are discussed. Additionally, materials that form the foundation in this study are discussed. Section 4.1.1 discusses the NSI dataset containing bilingual NSIs for text detection. Section 4.1.2 discusses the cropped Urdu character dataset used for recognizing Urdu characters. Section 4.1.3 discusses the cropped Urdu word dataset used for recognizing cropped Urdu words. Section 4.1.4 discusses the cropped English character dataset used for recognizing English characters. Section 4.1.5 covers the construction of a bilingual dataset from a unilingual dataset along with the techniques used for dataset augmentation and preprocessing.

#### 4.1.1. Natural Scene Images

Bilingual NSIs formed the first segment of the dataset and contained images from public spaces such as signboards, hoardings, banners, navigation boards, and banners containing Urdu and English text. The base dataset contained 945 images of bilingual NSIs [13]. This segment was augmented by employing various augmentation techniques. The proposed bilingual dataset contained 2835 bilingual NSIs, and dataset augmentation prevented model overfitting. This segment of the dataset enabled the reliable detection of Urdu and English (bilingual) text in NSIs.

#### 4.1.2. Cropped Urdu Characters

The second segment of the dataset contained 19,901 images of cropped Urdu characters along with their labels [13], extracted from the first segment of bilingual NSIs. To increase diversity and balance, we applied several augmentation techniques. The proposed bilingual dataset contained 59,703 images of cropped Urdu characters. The images were classified into 39 unique and distinct classes, with each class representing a unique character from the Urdu text. The number of instances in each class was different depending on the occurrence and the usage of the character in the Urdu language script. All images were fixed to a size of 48 × 46 pixels for training the deep learning model. This segment was used for recognizing cropped Urdu characters.

#### 4.1.3. Cropped Urdu Words

The third segment of the dataset contained 14,099 images of cropped Urdu words along with their labels [13], also extracted from the first segment of the bilingual NSIs. After augmentation, the resulting bilingual dataset contained 42,297 images of cropped Urdu words. The characters in the words were also classified into 39 unique and distinct classes. All the images were fixed to a size of 100 × 64 pixels to ensure a standardized input for training the deep learning model. This segment was used for recognizing cropped Urdu words.

#### 4.1.4. Cropped English Characters

This segment contained images of cropped English characters. The original base dataset [13] did not contain any cropped English character dataset. We contributed by manually cropping a total of 14,224 characters from the base dataset of bilingual NSIs. We also labeled this dataset manually. This segment was enhanced using various augmentation techniques, and the resulting bilingual dataset contained 42,672 images of cropped English characters. These characters were classified into 52 unique and distinct classes, with each class representing a unique character from the English text. All images were fixed to a size of 192 × 190 pixels. This segment of the dataset was used for the training and recognition of English characters.

#### 4.1.5. Dataset Creation and Preprocessing

A publicly available dataset, the Cursive Text dataset [13], was used as the base dataset in this study. This dataset is widely used for Urdu text recognition in natural scene images. The dataset provides a comprehensive set of real-world images with detailed ground truth annotations at the character, word, and line levels, making it a valuable resource for advancing research in Urdu OCR.

While popular multilingual benchmarks such as ICDAR MLT 2017 and MLT 2019 exist, they primarily focus on scripts such as Latin, Chinese, Devanagari, and Arabic. Notably, the Urdu script is not included in these benchmarks, which limits their applicability for evaluating bilingual systems involving Urdu and English. Given this, the Cursive Text dataset offers the most relevant and appropriate foundation for developing and testing Urdu–English recognition systems.

To adapt the dataset for bilingual recognition tasks, we extended it by extracting English text components from the original natural scene images. Additionally, we applied a range of augmentation techniques—such as scaling, rotation, contrast variation, and noise addition—to both Urdu and English samples. This enhanced dataset supported more robust learning and enabled meaningful evaluation of bilingual recognition models under varied conditions.

**Figure 6:**
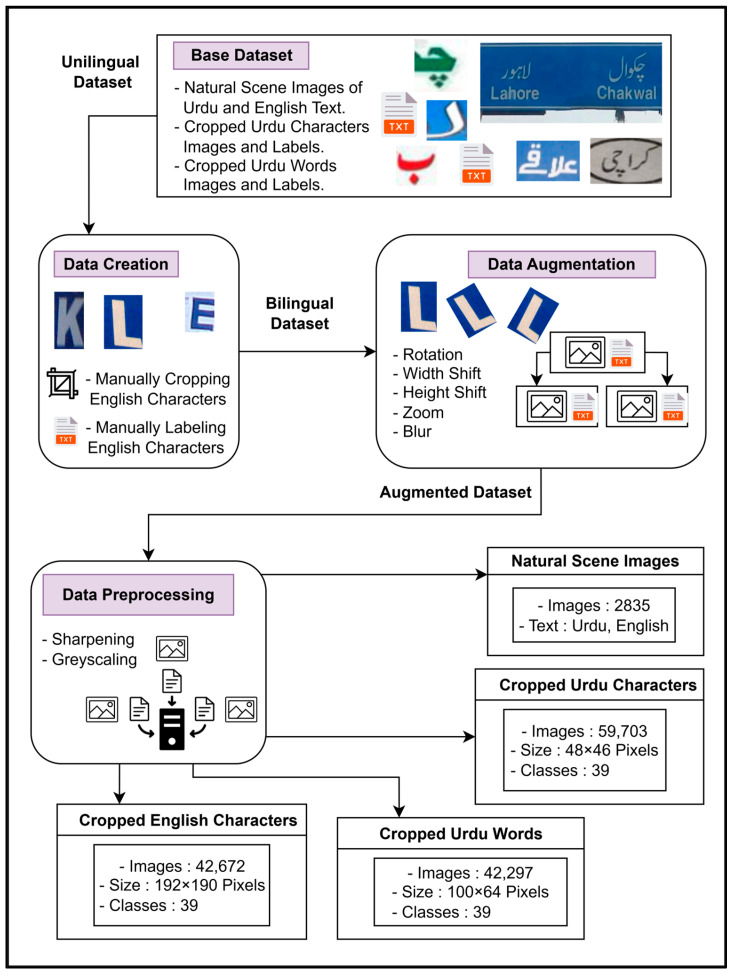
Dataset creation and preprocessing framework.

The dataset framework begins with the unilingual base dataset that was input in the data creation stage to obtain a bilingual dataset. The pipeline for the proposed dataset creation and preprocessing is as follows:

- In this framework, the preliminary base dataset [13] originally contains the following:
  ◦ Bilingual NSIs containing Urdu and English text;
  ◦ Images of cropped Urdu characters segmented from these NSIs and their labels;
  ◦ Images of cropped Urdu words segmented from these NSIs and their labels.
- This dataset was previously used to detect only Urdu text. It is enhanced for detecting bilingual text in the data creation stage.
  ◦ This stage involves manually segmenting the images of cropped English characters from the preliminary bilingual NSIs. The resulting dataset is used for training the model on the English model.
  ◦ Additionally, ground truth labels are manually created for this dataset containing cropped English characters.
  ◦ The unilingual dataset is finally converted into a bilingual dataset (Figure 6).
- The output segments of the data creation stage are fed as the input to the data augmentation stage. In this stage, the sizes of all four segments of the dataset are increased using augmentation techniques such as rotation, width shift, height shift, zoom, blur, and color adjustments. These augmentation techniques are carefully designed to preserve the inherent characteristics of both Urdu and English text and enhance the performance of the model in detecting bilingual text in NSIs.
- The output segments are also fed to the data preprocessing stage that is carefully designed to improve the quality of input segments. Preprocessing techniques such as sharpening and grayscale are applied for enhancing feature detection and minimizing background complexities. This stage is important for the accurate detection and recognition of bilingual text from NSIs.

The comparison of the number of images across different stages of this dataset framework is shown in Table 2.

### 4.2. Methods: Proposed Methodology

The proposed method was designed using state-of-the-art deep learning methods. Its pipeline comprised two modules: text detection and text recognition; these are discussed in Section 4.2.1 and Section 4.2.2, respectively. The pipeline for the proposed methodology is as follows:

- The pipeline begins with an image being fed into the text detection module that is used to localize the instances of text in the image. It also draws the boundaries of the text regions on the image.
- The text recognition module performs feature extraction of the cropped Urdu characters and words and cropped English characters separately. Then, sequence modeling and sequence decoding are performed.
- The output reflects the text recognized from the NSI and offers insights into the efficient functioning of the proposed pipeline.
  ◦ The predicted text is the primary output, which is a readable form of the text extracted by the model from the input NSIs.
  ◦ The text recognition rate is the secondary output that is a measure of the correctness of the predicted text.

**Figure 7:**
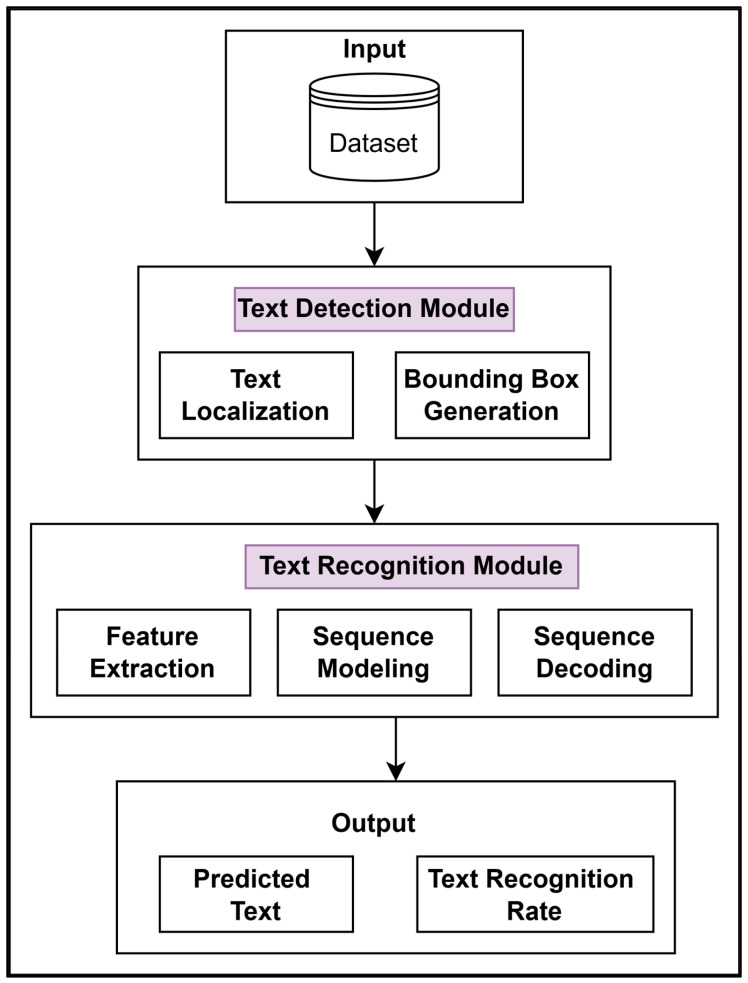
Pipeline for end-to-end bilingual text detection and recognition in NSIs.

The proposed methodology employed these systematic steps to achieve high accuracy in text recognition from complex and diverse NSIs. The aforementioned pipeline is shown in Figure 7.

#### 4.2.1. Text Detection Module

**Figure 8:**
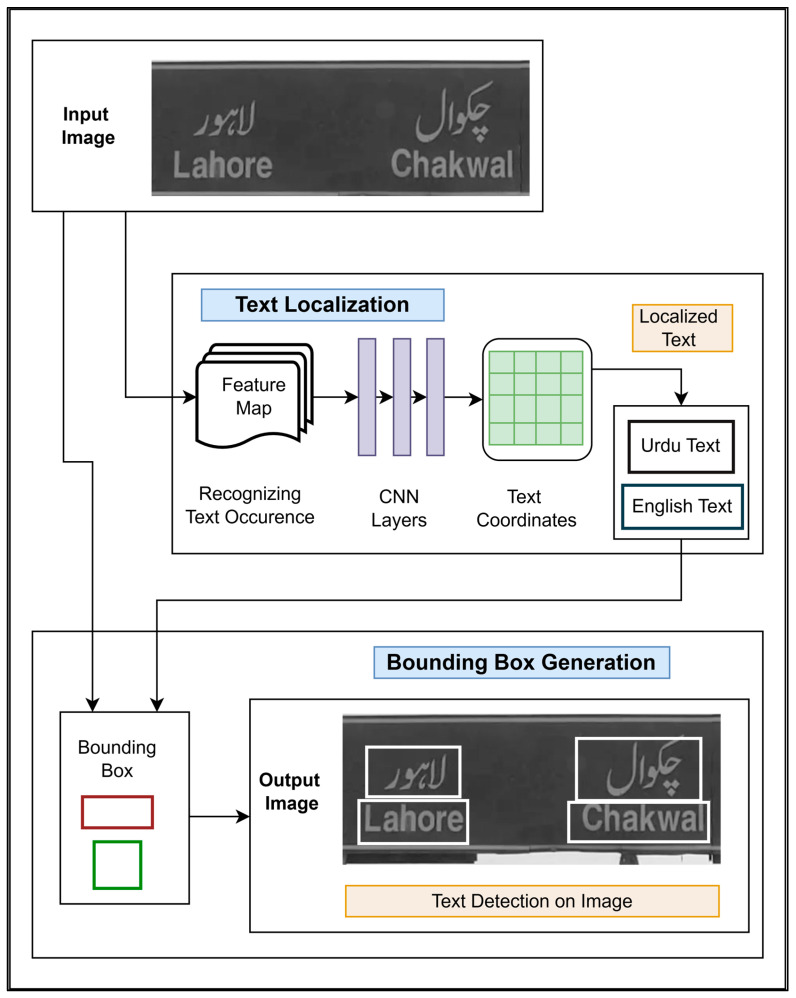
Text detection module with two main stages: the text localization stage, in which text coordinates (both Urdu and English) in the input image are identified, and the boundary box generation stage, in which bounding boxes are used to highlight the detected text on the image.

The preprocessed NSIs were fed as the input to the text detection module. This module extracted meaningful information from the images by separating text regions from the image background. It is shown in Figure 8.

In the text localization stage of the module, an input image is fed into the CNN layers of an optical text localization model. The occurrence of the text in the image is identified, and a feature map is created that is fed through the CNN layers trained to differentiate between Urdu and English text. These CNN layers analyze the feature map thoroughly to separate it into Urdu and English text coordinates. Thus, as an output, Urdu text and English text is localized separately.

In the bounding box generation stage, the output text coordinates are combined with the same input image; these are fed to a bounding box method that draws rectangular boxes around the detected text regions. The combined result of these two methods is used for text region detection. Thus, an output image is shown with bounding boxes drawn around detected text.

#### 4.2.2. Text Recognition Module

**Figure 9:**
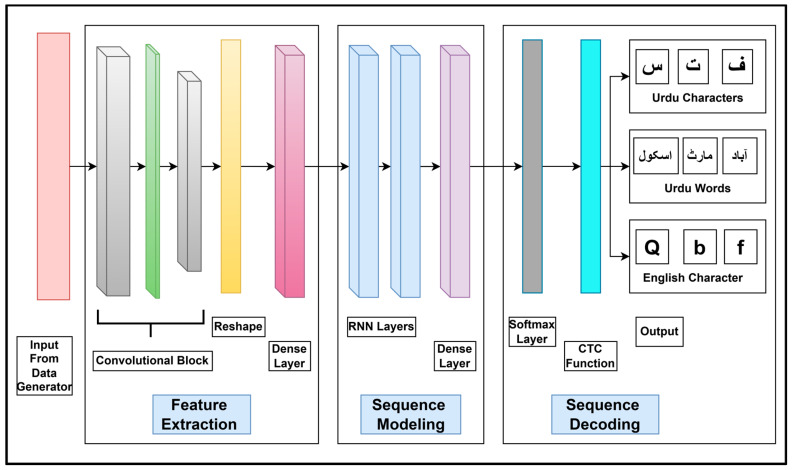
Text recognition module comprising three stages: feature extraction, sequence modeling, and sequence decoding. The layers involved in processing input data via convolutional blocks, the reshaping layer, dense layers, RNN layers, and Softmax and CTC functions for text decoding are also shown.

The text recognition module is categorized into three stages: feature extraction, sequence modeling, and sequence decoding (Figure 9).

As shown in Figure 9, a preprocessed input image along with the input labels are fed to a data generator that loads the image and labels. It further creates badges and assembles the inputs into required forms. The data generator is designed to preprocess the images as well as decode and encode labels according to the needs of the model.

The output from the data generator is fed to the feature extraction stage (Figure 9) that contains a convolutional block of CNN layers. These CNN layers are associated with Relu activations, followed by max pooling layers with different strides. Together, these layers extract spatial features from both Urdu and English text. The resulting spatial feature map is reshaped into a one-dimensional sequence using a reshape layer, simulating a temporal progression across the text. These sequential features are then passed through a dense layer for enhancement before being forwarded to the sequence modeling stage.

**Figure 10:**
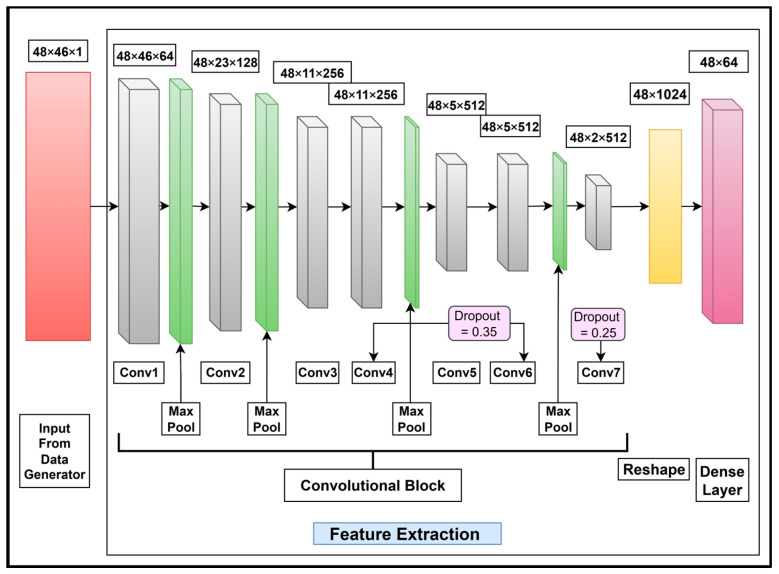
Feature extraction layers for Urdu character recognition based on the feature extraction stage in Figure 9. Additionally, the convolutional layers; the max pooling, dropout, and reshaping operations; and a dense layer specifically tailored for extracting features from cropped Urdu characters are shown.

The feature extraction stage follows a similar structure across all categories, with slight adjustments in the convolutional block configurations to suit Urdu character and word recognition and English character recognition. These feature extraction layers are described as follows:

The feature extraction layers for Urdu character recognition comprise seven fully connected layers with 3 × 3 filters for the CNN block of the feature extraction stage. The convolutional layers contain 64, 128, 256, 256, 512, 512, and 512 units. A max pooling layer of 1 × 2 stride is used after Conv1, Conv2, Conv4, and Conv6 only. A dropout layer with a rate of 0.35 is used with Conv4 and Conv6 and with a rate of 0.25 with Conv7. At the end of Conv7, a reshape layer with 1024 units and a dense layer of 64 units is used. The proposed feature extraction layers for Urdu character recognition are shown in Figure 10.

**Figure 11:**
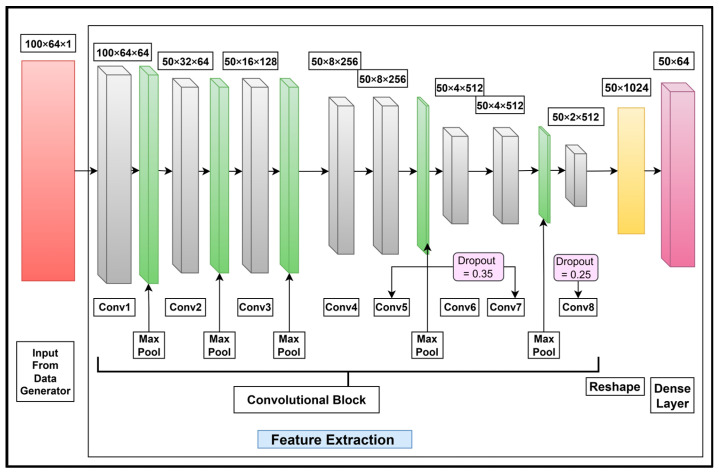
Feature extraction layers for Urdu word recognition based on the feature extraction stage in Figure 9. The convolutional layers; the max pooling, dropout, and reshaping operations; and a dense layer specifically tailored for extracting features from cropped Urdu words are also shown.

The feature extraction layers for Urdu word recognition are shown in Figure 11.

As shown in Figure 11, eight fully connected layers with 3 × 3 filters are used for the CNN block in the feature extraction stage. The convolutional layers contain 64, 64, 128, 256, 256, 512, 512, and 512 units. A max pooling layer of 2 × 2 stride is used after Conv1, and a max pooling layer of 1 × 2 stride is used after Conv2, Conv3, Conv5, and Conv7 only. A dropout layer with a rate of 0.35 is used with Conv5 and Conv7 and a layer with a rate of 0.25 is used with Conv8. At the end of Conv8, a reshape layer with 1024 units and a dense layer of 64 units are used.

**Figure 12:**
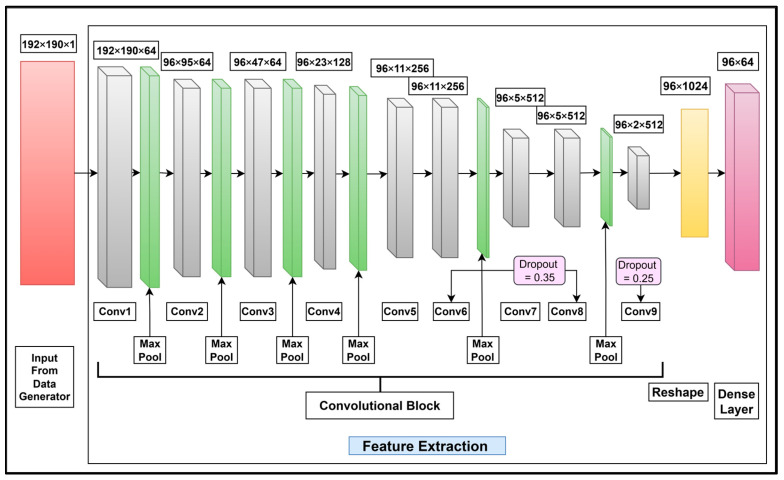
Feature extraction layers for English character recognition based on the feature extraction stage in Figure 9. The convolutional layers; the max pooling, dropout, and reshaping operations; and a dense layer specifically tailored for extracting features from cropped English characters are also shown.

The feature extraction layers for English character recognition comprise nine fully connected layers with 3 × 3 filters in the CNN block of the feature extraction stage. The convolutional layers contain 64, 64, 64, 128, 256, 256, 512, 512, and 512 units. A max pooling layer of 2 × 2 stride is used after Conv1 and a max pooling layer of 1 × 2 stride is used after Conv2, Conv3, Conv4, Conv6, and Conv8 only. A dropout layer with a rate of 0.35 is used with Conv6 and Conv8 and a layer with a rate of 0.25 is used with Conv9. At the end of Conv9, a reshape layer with 1024 units and a dense layer of 64 units are used. The proposed feature extraction layers for English character recognition are shown in Figure 12.

In the sequence modeling stage, illustrated in Figure 9, two stacked RNN layers receive the sequential features extracted during the feature extraction stage. These RNNs learn temporal dependencies within these sequential features and generate a refined sequence of high-level temporal encodings, often referred to as logits across time. This enables the model to capture the structure of a word more effectively than a CNN alone, especially for scripts like Urdu (cursive) where characters are connected and character shape varies based on position and adjoining ligatures. The output from the RNN layers is then passed through a dense layer whose number of units equals the number of target classes. For this stage, experiments are conducted using various RNNs and hidden units; the corresponding configuration results have been discussed in Section 6. The final output of this stage is fed to the sequence decoding stage.

In the sequence decoding stage, a Softmax activation layer is applied. The Softmax activation produces a sequential prediction over all target classes at each timestep, thus reflecting the model’s confidence across target classes. To decode these sequential predictions into the final transcription, a connectionist temporal classification (CTC) loss function is applied. A CTC loss function is specifically designed to determine the most likely transcription by considering all possible alignments between the predicted text sequence and the ground truth sequence. This CTC function is particularly effective in scenarios wherein the input and output sequences have variable lengths. During training, text alignment and transcription accuracy are evaluated and the loss is backpropagated. The CTC function helps to minimize this loss as it is particularly designed for sequential data. This model is compiled using an Adam optimizer with a learning rate of 0.001 and is evaluated based on the text recognition rate.

## 5. Evaluation Indicators and Their Significance

The model performance was evaluated based on recognition rates such as character recognition rate (CRR) and word recognition rate (WRR). These rates indicate the ability of the models to recognize the given unique and unseen samples of data. These are discussed in Section 5.1 and Section 5.2, respectively.

### 5.1. CRR

CRR is used to measure the system performance at the character level. It evaluates how well the system has predicted characters in character recognition tasks. It is also used in word recognition tasks to measure the accurately predicted characters in a word. This metric is used in Urdu and English character recognition tasks.

CRR can be calculated as follows:

$$CRR=\frac{\sum_{i=0}^{n}\sum_{j=1}^{Min\left(X_{i},Y_{i}\right)}\partial \left(A_{i}\left[j\right],P_{i}\left[j\right]\right)}{\sum_{i=1}^{N}Max\left(X_{i},Y_{i}\right)}\times 100,$$

where

- *X_i_* is the number of characters in the ith actual label;
- *Y_i_* is the number of characters in ith predicted label;
- *A_i_[j]* is the ith actual label of the jth letter;
- *P_i_[j]* is the ith predicted label of the jth letter;
- *δ(A_i_[j]*, *P_i_[j])* is

$$\partial \left(A_{i}\left[j\right],P_{i}\left[j\right]\right)=\left\{\begin{array}{c} 1,\;\;if\;A_{i}\left[j\right]=P_{i}\left[j\right]\\ 0,\;\;otherwise \end{array}\right..$$

- *Min (X_i_,Y_i_)* denotes the minimum number of characters in the actual and predicted labels to be compared by the system.
- *Max (X_i_,Y_i_)* denotes the maximum number of characters in the actual and predicted labels that facilitate accurate comparison.

CRR is determined by taking the total of correctly predicted characters of all predicted labels and then dividing it with the maximum possible number of characters in all actual labels. A higher CRR indicates better model performance and vice versa.

### 5.2. WRR

The WRR is used to measure the model performance at the word level. It evaluates how well the system predicts whole words in word recognition tasks. Unlike CRR, this metric is more realistic because it considers the accuracy of the entire word and is therefore used in the Urdu word recognition task.

WRR is calculated as follows:

$$WRR=\frac{1}{N}\sum_{i=1}^{N}\partial \left(A_{i},P_{i}\right)\times 100,$$

where

- *N* is the total number of labels;
- *A_i_* is the ith actual label;
- *P_i_* is the ith predicted label;
- *δ(A_i_, P_i_)* is

$$\partial \left(A_{i},P_{i}\right)=\left\{\begin{array}{cc} 1, & \;\;if\;A_{i}=P_{i}i\\ 0, & \;\;otherwise \end{array}\right.$$

WRR is calculated by taking a total of correctly predicted words of all predicted labels and then dividing it with the total number of words in all actual labels. A higher WRR indicates the enhanced prediction performance of the model and vice versa.

CRR and WRR are important for determining the performance of text recognition systems. WRR reflects the ability of a model to predict complete words accurately. In real-world applications, accomplishing high WRRs is vital for confirming that the model can reliably predict full text. CRR is suitable for a better-quality prediction because it measures how well models recognize individual characters. High CRRs are equally significant for reducing blunders that may arise from incorrect or misidentified characters in complex text, i.e., Urdu text in our case. Thus, both metrics were used and text recognition models were enhanced and evaluated, particularly for complex scripts such as Urdu in NSIs with adverse factors.

## 6. Results

Herein, the results of this study are presented and discussed. Section 6.1 covers experiments pertaining to the sequence modeling stage of the text recognition module containing different RNNs and their hidden units. Section 6.2 covers the training and validation accuracy and loss of the best models. Section 6.3 discusses ablation studies of the best models. The proposed models were compared with existing models, as discussed in Section 6.4.

A system with a 64-bit processor, 8 GB of RAM, and a 500 GB SSD was used for experiments. Keras [45], Numpy [46], and Panda [47] libraries were used. The dataset was divided as follows: 70% for training, 20% for validation, and 10% for testing; it contained unique images. The total number of images was split across these sets, as shown in Table 3.

The set split ratio was well suited for this large dataset and the complex task of analyzing NSIs and multiple scripts. Using the training data, the model learned complex patterns from multiple scripts in NSIs. The validation set was used for fine-tuning hyperparameters and monitoring model overfitting during training. The training dataset was used for evaluating model generalizability on unseen data. This split ratio helped to achieve reliable, optimized, and high model performance for the detection and recognition of Urdu and English text in NSIs.

### 6.1. Model Performance Under Different RNNs and Hidden Units

Experiments were conducted for the CRNN model, and results were obtained under different RNNs and hidden units in terms of character and word recognition rates. The model that yielded the best results was selected for ablation studies. Table 4 summarizes the recognition rates obtained in each experiment.

A gated recurrent unit (GRU), bidirectional gated recurrent unit (BGRU), long short-term memory (LSTM), bidirectional long short-term memory (BLSTM), and two combinations of RNN hidden units, i.e., 256 and 512, were used in the experiments. GRU-512 yielded better results than GRU-256. In addition, BGRU-512 yielded better results than BGRU-256. Similarly, LSTM-512 and BLSTM-512 yielded better recognition rates than LSTM-256 and BLSTM-256, respectively. Bidirectional models achieved higher recognition rates than unidirectional models. Compared to GRU models, LSTM models achieved better results. However, BGRU models achieved better rates than LSTM models. These findings indicated that BLSTM models achieved the best results among all models. In summary, BGRU-512 and BLSTM-512 outperformed the other models. Both of these models were bidirectional and used 512 hidden units. The second-best model BGRU-512 achieved an Urdu CRR of 97.8%, Urdu word recognition rate (WRR) of 96.1%, and English CRR of 96.5%. The best model BLSTM-512 achieved a remarkable Urdu CRR of 98.5%, Urdu WRR of 97.2%, and English CRR of 99.2%.

### 6.2. Accuracy and Loss Trends of Proposed Models

**Figure 13:**
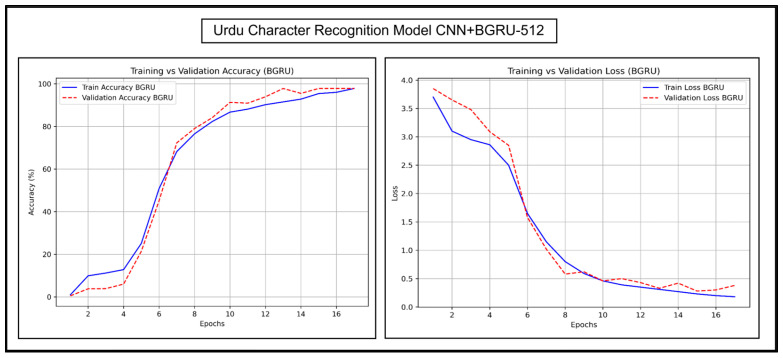
Accuracy and loss trends of Urdu character recognition for CNN+BGRU-512.

**Figure 14:**
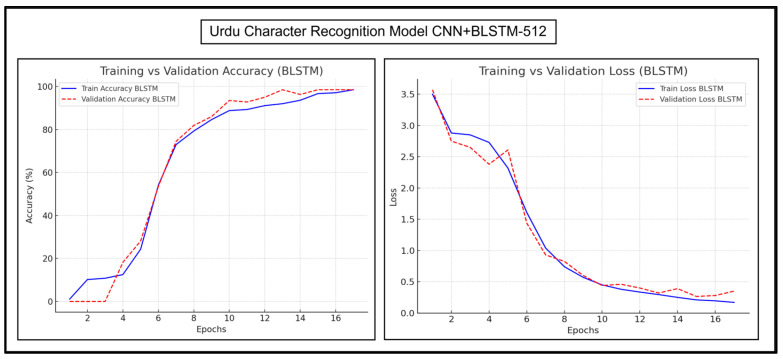
Accuracy and loss trends of Urdu character recognition for CNN+BLSTM-512.

The accuracy and loss trends of CNN+BGRU-512 and CNN+BLSTM-512 are discussed in detail herein. Their accuracy and loss graphs for experimentations on Urdu character recognition, Urdu word recognition, and English character recognition have been presented and analyzed. Figure 13 and Figure 14, respectively, show the Urdu CRRs of both of these models.

Figure 13 shows that the training curves of CNN+BGRU-512 appear smooth and similar to the validation curves, indicating minimal overfitting. The validation and training losses decrease steadily, indicating reliable learning. The validation loss is slightly higher in the beginning epochs. There is a fluctuation in epoch 5, and the curves then stabilize quickly with minor fluctuations in later epochs.

Figure 14 shows that CNN+BLSTM-512 also has a smooth and upward training curve. The validation curve is slightly ahead of the training curve, which makes it more generalized than CNN+BGRU-512. The validation and training losses of CNN+BLSTM-512 first decrease considerably. The validation loss is slightly lower than the training loss initially, which indicates more effective learning than CNN+BGRU-512. CNN+BLSTM-512 also maintains a more stable loss curve in later epochs. Overall, the loss curves of both models reached a low level, ensuring high accuracies in the accuracy graph. Their accuracy reached 80% around epoch 8, proving their robustness.

**Figure 15:**
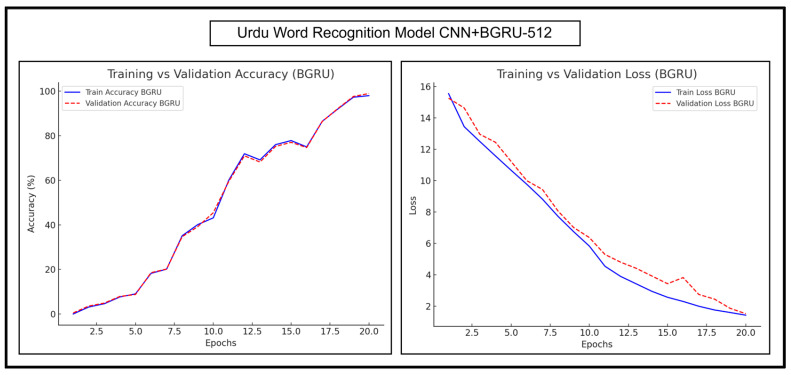
Accuracy and loss trends of CNN+BGRU-512 during Urdu word recognition.

**Figure 16:**
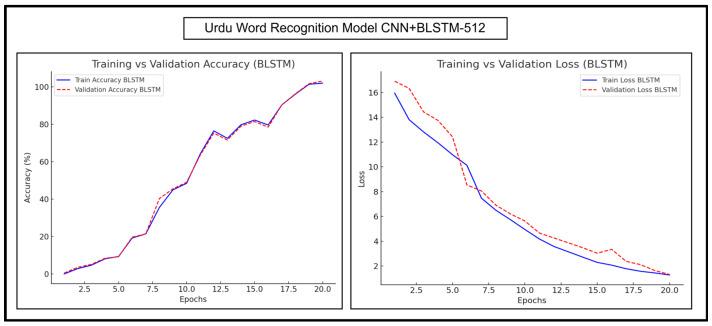
Accuracy and loss trends of CNN+BLSTM-512 during Urdu word recognition.

The trends for Urdu word recognition experimentations of CNN+BGRU-512 and CNN+BLSTM-512 are shown in Figure 15 and Figure 16, respectively.

Figure 15 shows that the training and validation curves of CNN+BGRU-512 were almost parallel and had a very steady increase, indicating minimal overfitting. These curves showed fluctuations, indicating that the model was exploring different patterns in the complex dataset and fine-tuning its parameters. The training and validation losses of CNN+BGRU-512 gradually decreased as the number of epochs increased, indicating the increasing stability of the learning process. A minor increase in the validation loss was observed at epoch number 16; however the model converged itself to the minimum loss.

As shown in Figure 16, the training and validation accuracy curves of CNN+BLSTM-512 increased with minor fluctuations as the number of epochs increased. Both curves were closely aligned, except for a minor increase in the validation curve at epoch number 8. The validation loss of CNN+BLSTM-512 was slightly higher in the initial epochs and showed a minor decline at epoch number 6; however, it stabilized and maintained a trend similar to that of the training loss in the later epochs. Overall, both CNN+BLSTM-512 and CNN+BGRU-512 models exhibited strong consistency in terms of the generalization ability, and the loss trend of BLSTM was slightly better than that of BGRU; therefore, BLSTM offered higher accuracy.

**Figure 17:**
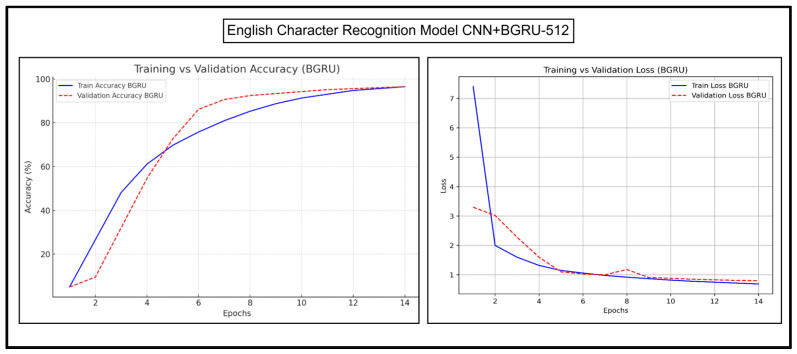
Accuracy and loss trends of CNN+BGRU-512 during English character recognition.

**Figure 18:**
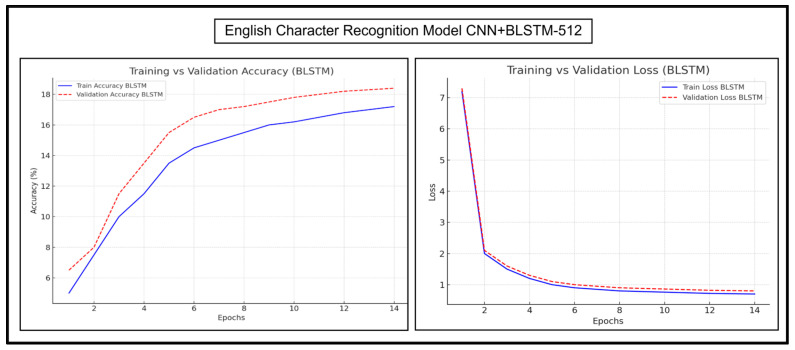
Accuracy and loss trends of English character recognition for CNN+BLSTM-512.

Figure 17 and Figure 18 show the results of English character recognition by CNN+BGRU-512 and CNN+BLSTM-512, respectively.

As shown in Figure 17, the training accuracy curve of CNN+BGRU-512 rapidly rose in the early epochs. The validation accuracy curve followed the same trend; however, the validation accuracy was slightly lower in the early epochs and slightly higher in later epochs. Both curves converged slowly, showing that the model stabilized, and its performance did not change much in later epochs. The training loss of CNN+BGRU-512 declined sharply in the initial epochs due to fast convergence. The validation loss was slightly higher than the training loss. It slightly decreased at epoch number 5 and suddenly increased at epoch number 6, but quickly stabilized with the training curve. Both loss curves quickly converged at later epochs, showing a stabilized learning phase.

Figure 18 shows that the training and validation accuracy curves of CNN+BLSTM-512 increase steadily in the upward direction. The validation accuracy curve is slightly higher than the training curve throughout the epochs, indicating the strong generalization of the model. The accuracy levels are relatively lower than CNN+BGRU-512 in the initial epochs but improve consistently, resulting in better generalization. CNN+BLSTM-512 also shows smooth training and validation loss curves, indicating its strong generalization. The validation loss curve is slightly higher than the training curve but in parallel to the training curve. Overall, both models perform well, but BLSTM exhibits a more stable and gradual learning process, with a stronger generalization and better results.

### 6.3. Ablation Studies of Proposed Models

CNN+BGRU-512 and CNN+BLSTM-512 were further used in ablation studies to analyze the performance of their components, and the importance of model components was highlighted. The first set of experiments was conducted on CNN+BGRU-512, and results were recorded as CRRs and WRRs (Table 5).

As shown in Table 5, the text recognition rates decrease when any of the model components are removed. BGRU-512 with augmentation, a dense layer, and BLSTM achieved better results. It was well trained on Urdu and English text. The second set of experiments was conducted on CNN+BLSTM-512, and the results were recorded as CRRs and WRRs (Table 6).

As shown in Table 6, the text recognition rates decrease when any of the model components are removed. A high recognition rate indicates accurate recognition, which is important for applications that require high precision. BLSTM-512 with augmentation, a dense layer, and BLSTM has achieved better results, indicating that it is well trained on Urdu and English text.

### 6.4. Analysis of Error Cases

In this section, the analyses of error cases in this recognition pipeline are discussed. These failure cases provide valuable insights and a thorough understanding of where and why the system encounters difficulties.

**Figure 19:**
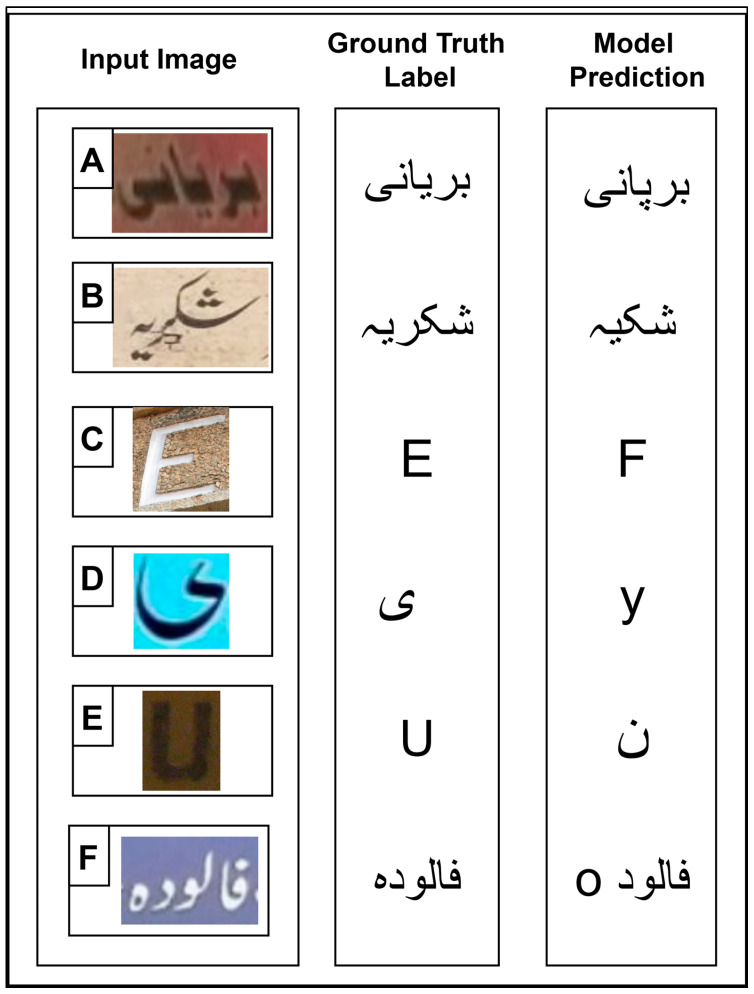
Sample of NSIs obtained from our proposed dataset: (**A**) Misclassification due to visually similar Urdu characters; (**B**) Error caused by nontext objects; (**C**) Low resolution input leading to incorrect prediction; (**D**) Confusion between Urdu and English characters; (**E**) Bilingual misclassification between Urdu and English; (**F**) Script orientation error in bilingual text. All samples illustrate error cases analysis, with their ground truth labels and the proposed model (CNN+BLSTM-512) predictions.

As shown in Figure 19 image “A”, a misclassification occurred due to visually similar Urdu characters. The ground truth label was “Biryani”, while the model incorrectly predicted “Birpani”, replacing “Paay” with “Yaay”. These characters share similar ligatures, differing only by the placement of dots, which can be challenging for models to distinguish, especially in cases where the dots are faint or blended with the background. Moreover, Figure 19 image “B” depicts an error caused by nontext objects. The ground truth label was “Shukriya”, but the model predicted “Shukiya”, failing to recognize the “Raay” character. A faint mark near the “Raay” may have contributed to its omission. This type of issue typically arises in noisy NSIs, due to artifacts like smudges, overlays, and camera noise. In Figure 19 image “C”, the effect of low-resolution input is observed. The ground truth label was “E”, yet the model incorrectly predicted it as “F”. Such errors are common, especially when character boundaries are not clear due to blurring, motion, or orientation. Characters with similar shapes, such as “E” and “F”, become indistinguishable under these conditions.

Furthermore, Figure 19 presents several notable bilingual misclassification cases. As shown in Figure 19 image “D”, there is a confusion between the Urdu character “Yeh” and the English lowercase character “y”. This visual closeness can easily mislead the recognition model, particularly when Urdu is written in casual, cursive styles on public boards or posters. Also, in Figure 19 image “E”, the ground truth label is an English uppercase character “U” but the model predicts it as an Urdu character “Nuun”. This error arises due to many factors, such as the curved part of both characters and the weak contextual cues in bilingual environments.

An error case in Figure 19 image “F” demonstrates a unique bilingual error influenced by script orientation. The Urdu word “Falooda” is written in its correct right-to-left orientation. However, the model mistakenly inserts an English character “o” at the leftmost position, and the rest of the Urdu word “Falood” at the right-most position, with a space in between. This misrecognition highlights a critical challenge in bilingual NSIs: confusion arising from conflicting text orientation. Urdu is inherently a right-to-left cursive script, while English follows a left-to-right non-cursive structure. In natural scene settings, especially when text from both languages coexists, the recognition model may struggle to correctly align character sequences. Figure 19 shows the sample of our dataset, along with its ground truth labels and the proposed model (CNN+BLSTM-512) predictions.

These scenarios emphasize the importance of handling ligature-level subtleties, nontext objects, blurriness, and resolution-aware recognition in noisy NSIs. Moreover, the system’s bilingual sensitivity makes the recognition task more challenging, due to script-level similarities, contextual cues, and orientation mismatches between Urdu and English text. Despite these complexities, our proposed recognition model demonstrates strong robustness and adaptability, effectively handling such variations and maintaining a high level of performance across diverse error scenarios.

### 6.5. Comparison of Proposed Models with Existing Models

Recent studies [22,23,24] performed Urdu character recognition using HOG, CNN, and MLFF, achieving recognition rates of 73.0%, 88.6%, and 93.0%, respectively. CRNN was also used for experiments on Urdu character and word recognition, achieving a 95.7% CRR and an 87.1% WRR, respectively [25].

Table 7 shows the results of models obtained using a publicly available dataset [13] in terms of Urdu CRR and WRR. Existing studies [22,23,24,25] have also evaluated Urdu CRR and WRR on the same dataset [13], making these results directly comparable. English CRR is reported only for the proposed models because the original dataset [13] included bilingual NSIs (Urdu and English text) and did not include cropped English characters. These characters were manually prepared as part of our extended evaluation in the created bilingual dataset.

Both the proposed models were trained to perform Urdu character and word recognition. CNN+BGRU-512 achieved an Urdu CRR of 97.2% and Urdu WRR of 91.6% on the Cursive Text dataset [13]. CNN+BLSTM-512 achieved a remarkable Urdu CRR of 98.1% and Urdu WRR of 93.4% on the dataset.

As shown in Table 6, CNN+BGRU-512 achieved 24.2%, 8.6%, and 4.2% improvements in Urdu CRR compared with existing models HOG [22], CNN [23], MLFF [24] on the dataset [13]. Similarly, CNN+BLSTN-512 achieved 25.1%, 9.5%, and 5.1% improvements. CNN+BLSTM-512 achieved better results than CNN+BGRU-512 [13].

As shown in Table 6, CNN+BGRU-512 achieved an improvement of 1.5% in Urdu character recognition and 4.5% in Urdu word recognition compared to using the existing model CRNN [25] on the dataset [13]. Similarly, CNN+BLSTM-512 achieved improvements of 2.4% in Urdu character recognition and 6.3% in Urdu word recognition as compared to the latest research [25] on the dataset [13]. Furthermore, the dataset [13] originally contained bilingual NSIs containing both Urdu and English text; however, previous studies used only Urdu text from these NSIs. In contrast, we used English text and CNN+BGRU-512 and CNN+BLSTM-512 achieved English CRRs of 92.6% and 93.5%, respectively.

## 7. Discussion

The pipeline used in this research, CNN-RNN-CTC, is trained on an enhanced bilingual dataset. The CNN is customized for Urdu characters, Urdu words, and English characters to extract their features. These extracted features are learned through RNNs. For this purpose, experiments are conducted using different RNN types and RNN hidden units. The results demonstrate that using bidirectional RNNs with larger hidden units significantly improves recognition rates. In particular, the second-best model CNN+BGRU-512 and the best model CNN+BLSTM-512 outperform and achieve better results for Urdu CRR, Urdu WRR, and English CRR. This pipeline handles the complexities of both Urdu and English scripts within the same NSI.

Furthermore, ablation studies confirm the critical role of each component of the pipeline, such as data augmentation, dense layers, and bidirectional RNN layers. It is clearly demonstrated that after applying augmentation in the dataset, accuracy rates for the pipeline are improved. The removal of dense layers and bidirectional layers leads to a decline in recognition rates in both models (BGRU and BLSTM). The two best proposed models, CNN+BGRU-512 and CNN+BLSTM-512, are compared with the existing approaches HOG [22], CNN [23], MLFF [24], and CRNN [25], all evaluated on the same benchmark Cursive Text dataset [13]. The proposed models demonstrate superior recognition rates over these existing methods. Thus, this research strengthens the field by tackling bilingual text detection and recognition challenges in diverse NSIs, laying a foundation for future multilingual natural scene understanding applications.

## 8. Conclusions

Herein, a pipeline was developed for the detection and recognition of Urdu and English text in NSIs. Our models incorporated a text detection module for localizing text instances in NSIs with adverse factors. The proposed models successfully recognized Urdu characters and words despite their cursive nature along with English characters. These models comprised customized CNNs for the feature extraction of Urdu characters and words and English characters. Models with different RNNs and hidden units were evaluated. Two best models were used in ablation studies to analyze the contribution of model components to overall performance. To date, no such models have been developed that detect English and Urdu with a high accuracy. The proposed models outperformed existing models in terms of the CRR and the CRR of bilingual text.

Unilingual datasets have been used in previous studies for text recognition; however, bilingual datasets have not been developed yet. To this end, a unilingual dataset was converted into a bilingual dataset herein, and English characters from bilingual NSIs were manually cropped and labeled. The proposed models were successfully trained on this custom-made dataset. This dataset was augmented to prevent model overfitting. This study highlights our commitment to solving challenges associated with bilingual text detection and recognition in NSIs containing Urdu and English text.

In the future, we plan to capture more NSIs to increase the training dataset size and enhance the model performance. This will ultimately increase the text recognition rate, making the proposed models more reliable. This research focused on recognizing Urdu characters and words and English characters and considered the challenges associated with character recognition. Future research will include English word recognition, further improving the models’ capabilities and enabling the creation of a robust text detection and recognition pipeline. Additionally, large language models (LLMs) have recently shown strong abilities in understanding both text and images. In the future, we plan to compare our model with these LLMs on OCR tasks, especially for multilingual and low-resource scripts like Urdu. We also aim to explore whether using or fine-tuning such models can improve results or add value alongside our current approach.

## Figures and Tables

**Table 1:** Methodology, datasets, and languages used in previous studies and their limitations.

Reference	Methodology	Dataset	Language (s)	Limitations
[33]	Maximally Stable Extremal Regions	ICDAR2001, ICDAR2002	English	Struggles with varying luminous conditions and cursive text
[34]	Laplacian Component Analysis	Hua’s dataset, MRRC, MSRA	English and Chinese	Lacks robustness for bilingual text with different orientations as well as with cursive text recognition
[35]	Histogram of Oriented Gradients	Custom dataset	English, Chinese, and Bengali	Manual feature extraction; time-consuming; and complex structure
[36]	Differentiation Binarization	MSRA-TD500	English and Chinese	Issues with overlapping objects, detailed textures, and cluttered backgrounds; struggles with cursive text recognition
[37]	Maximally Stable Extremal Regions	KAIST, COCO, CTW1500, CVSI, ICDAR	English, Chinese, and Korean	Challenges in the presence of lighting changes and lacks robustness for bilingual text with different orientations
[38]	CNN	Custom dataset	Gurumukhi	Limited to Gurumukhi script and cannot generalize to other scripts or conditions
[39,40]	Attention Mechanism	IIIT5k, SVT, ICDAR	English, Chinese, and Hindi	Sensitive to attention drifts; accuracy depends on precise attention alignment; and unilingual focus
[41,42,43]	Neural Networks	Benchmark datasets	English and ICDAR Languages	High sensitivity to text variability and alignment issues; struggles with pattern distortions and curved text
[21]	Recurrent CNN	Custom dataset	Urdu	Dataset comprises Urdu printed text manually pasted on images
[22]	Histogram of Oriented Gradients	Custom dataset	Urdu	Manual feature extraction and classifier dependency
[23]	CNN	Custom dataset	Urdu	Unilingual focus; struggles with complex cursive word recognition
[24]	Multilevel Feature Fusion	Custom dataset	Urdu	Focuses on isolated Urdu characters
[25]	Convolution Recurrent Neural Network	Custom dataset	Urdu	Limited dataset and lower accuracy compared to proposed model
[26]	Rank-1 Tensor Decomposition	Char74, ICDAR	English	Limited to English only and requires robust evaluation datasets
[27]	CNN	Char74k, ICDAR	English	Unilingual focus; lacking robustness under complex real-world NSIs
[28]	VGG-16	ICDAR 2017 bilingual dataset	Urdu and Arabic	Focus on only cursive text; lacking robustness for bilingual texts with different orientations
[29]	CNN	DLL-TraffSiD	Urdu and English	Small dataset; prone to overfitting; limited scalability
[32]	Artificial Neural Network	Custom dataset	Urdu and English	Dependent on training data quality; struggles with font variations; has lower accuracy compared to the proposed model

**Table 2:** Comparison of the number of images across different stages from the preliminary base dataset [13] to the proposed dataset after data creation, augmentation, and preprocessing.

Reference	Natural Scene Images	Cropped Urdu Characters	Cropped Urdu Words	Cropped English Characters
Preliminary unilingual base dataset [13]	945	19,901	14,099	-----
Proposed bilingual dataset (after data creation stage)	945	19,901	14,099	14,224
Proposed bilingual dataset (after data augmentation and data preprocessing stages)	2835	59,703	42,297	42,672

**Table 3:** Number of images in the proposed dataset and training, validation, and testing datasets.

Dataset	NSIs	Cropped Urdu Characters	Cropped Urdu Words	Cropped English Characters
Proposed dataset	2835	59,703	42,297	42,672
Training dataset	1985	41,792	29,608	29,871
Validation dataset	567	11,941	8459	8534
Test dataset	283	5970	4230	4267

**Table 4:** Experimental results of the CRNN model with different RNNs and hidden units.

No.	Model	RNN Type	RNN Hidden Units	Urdu CRR (%)	Urdu WRR (%)	English CRR (%)
1	CNN+RNN	GRU	256	85.4	87.2	87.6
2	CNN+RNN	GRU	512	85.8	83.7	89.4
3	CNN+RNN	BGRU	256	91.4	89.3	94.5
**4**	**CNN+RNN**	**BGRU**	**512**	**97.8**	**96.1**	**96.5**
5	CNN+RNN	LSTM	256	88.7	87.4	89.1
6	CNN+RNN	LSTM	512	89.6	88.3	90.5
7	CNN+RNN	BLSTM	256	98.2	96.4	99.2
**8**	**CNN+RNN**	**BLSTM**	**512**	**98.5**	**97.2**	**99.2**

**Table 5:** Ablation study of components of CNN+BGRU-512.

Model	Augmentation	Dense Layer	BGRU Layers	Urdu CRR (%)	Urdu WRR (%)	English CRR (%)
CNN+BGRU-512	Omitted	Absent	Present	89.9	84.1	90.4
CNN+BGRU-512	Omitted	Present	Absent	89.4	85.3	90.1
CNN+BGRU-512	Omitted	Present	Present	97.2	91.6	92.6
CNN+BGRU-512	Applied	Absent	Present	93.3	91.9	93.1
CNN+BGRU-512	Applied	Present	Absent	94.1	92.2	93.5
**CNN+BGRU-512**	**Applied**	**Present**	**Present**	**97.8**	**96.1**	**96.5**

**Table 6:** Ablation study of components of CNN+BLSTM-512.

Model	Augmentation	Dense Layer	BLSTM Layers	Urdu CRR (%)	Urdu WRR (%)	English CRR (%)
CNN+BLSTM-512	Omitted	Absent	Present	90.4	86.1	91.2
CNN+BLSTM-512	Omitted	Present	Absent	90.1	86.6	90.5
CNN+BLSTM-512	Omitted	Present	Present	98.1	93.4	93.5
CNN+BLSTM-512	Applied	Absent	Present	95.3	92.2	96.3
CNN+BLSTM-512	Applied	Present	Absent	96.1	94.3	96.7
**CNN+BLSTM-512**	**Applied**	**Present**	**Present**	**98.5**	**97.2**	**99.2**

**Table 7:** Comparison of recognition performance between existing models and our proposed models using the Cursive Text dataset [13].

Reference	Methods	Urdu CRR (%)	Urdu WRR (%)	English CRR (%)
[22] M. Pickering	HOG	73.0	-----	-----
[23] K. Shafi	CNN	88.6	-----	-----
[24] A. A. Chandio	MLFF	93.0	-----	-----
[25] A. A. Chandio	CRNN	95.7	87.1	-----
**Proposed model CNN+BGRU-512 (second best)**	**CRNN**	**97.2**	**91.6**	**92.6**
**Proposed model CNN+BLSTM-512 (best)**	**CRNN**	**98.1**	**93.4**	**93.5**

English CRR is reported only for the proposed models as the original dataset [13] did not include cropped English characters; these were manually prepared for the extended evaluation.

## Data Availability

The dataset used in this study is publicly available at https://doi.org/10.1016/j.dib.2020.105749.
